# Alleles associated with physical activity levels are estimated to be older than anatomically modern humans

**DOI:** 10.1371/journal.pone.0216155

**Published:** 2019-04-29

**Authors:** Ayland C. Letsinger, Jorge Z. Granados, Sarah E. Little, J. Timothy Lightfoot

**Affiliations:** Department of Health and Kinesiology, Texas A&M University, College Station, TX, United States of America; Universitat Pompeu Fabra, SPAIN

## Abstract

The purpose of this study was to determine the estimated mutation age and conservation of single-nucleotide polymorphisms (SNPs) associated with physical activity (PA) in humans. All human SNPs found to be significantly associated with PA levels in the literature were cross-referenced with the National Heart, Lung, and Blood Institute’s Grand Opportunity Exome Sequencing Project to find estimated African-American (AA) and European-American (EA) mutation age. As a secondary measure of mutation age, SNPs were searched for in Hawk’s mutation age prediction database which utilizes linkage equilibrium. To determine conservation among hominids, all SNPs were searched in the University of California, Santa Cruz Genome Browser, which contains Neanderthal and chimpanzee reference genomes. Six of the 104 SNPs associated with PA regulation were exon-located missense variants found in *IFNAR2*, *PPARGC1A*, *PML*, *CTBP2*, *IL5RA*, and *APOE* genes. The remaining 98 SNPs were located in non-protein coding regions. Average AA and EA estimated mutation age of the exon-located SNPs were 478.4 ± 327.5 kya and 542.1 ± 369.4 kya, respectively. There were four selective sweeps (suggestive of strong positive selection) of SNPs in humans when compared to Neanderthal or chimpanzee genomes. Exon-located PA candidate SNPs are older than the hypothesized emergence of anatomically modern humans. However, 95% of PA associated SNPs are found in intron and intergenic location. Across all SNPs, there seems to be a high level of conservation of alleles between humans, Neanderthals, and chimpanzees. However, the presence of four selective sweeps suggests there were selection pressures or drift unique to *Homo sapiens* that influenced the development of mutations associated with PA regulation.

## Introduction

While the benefits of physical activity (PA) are well known [1,2], a study of 7176 participants utilizing accelerometers [3] reported less than 3.5% of adults met the United States daily PA guideline of at least 30 minutes of daily moderate PA [4]. This level of physical inactivity among most humans has been shown to cause and exacerbate many diseases and increase rates of early mortality [5,6]. However, our closest primate relatives manage the same PA level as the most inactive humans, but seemingly do not suffer similar health consequences [7]. It seems as there is a physiological need for humans to engage in PA that is not shared with close relatives. Thus, understanding the biological factors responsible for increasing the likelihood of participating in PA is critical to avoid associated hypokinetic diseases and their impact on human health. In this article, PA refers to the conscious engagement of locomotion via muscle contraction. Thus, PA regulation refers to the biological factors that result in the engagement or disengagement of PA.

Substantial evidence indicates the regulation of PA levels in adult humans is heritable, as shown by over 45 twin and population studies [8]. In spite of the numerous studies in this area, there is still minimal agreement regarding which genetic mechanisms are involved due to the inherent complexity of PA regulation [8,9] as well as the many environmental factors [10] that may have genetic interactions. To date, there are few genome-wide association studies (GWAS) in humans that link the time spent engaged in PA with genomic variants. The available PA GWAS have generated a number of significant single-nucleotide polymorphisms (SNPs) at multiple genomic locations associated with PA, yet there is no particular region that is considered to be responsible for the majority of the variance in PA [8,9,11]. Due to this ambiguity, parsing out which of these regions are important and how/if they regulate PA requires novel approaches.

Predicting the emergence and spread of genetic regulators of PA in specific time eras could help identify potential environmental factors that would have served as selection pressures. For example, approximately 11,000 years ago (11 kya; [12]) agriculture rapidly spread, requiring a fivefold increase in duration of PA for farmers on a daily basis [13]. A farmer who did not meet the needs for increased PA could not produce enough food to support fecundity and subsequent population growth. This agricultural-induced increase in daily PA could have induced genetic alterations favoring increased PA adherence in some population by altering preferred metabolic pathways [13]. However, once farming methods of a region could produce larger yields, the greater percentage of associated humans no longer needed to engage in the same amount of PA. While this example is limited in its explanatory value due to variation in agriculture progress between regions of the world, estimating the age of the identified PA-related genomic variants could provide initial information of when and how the current state of human PA regulation could have been established. Alternatively, potential mechanisms regulating PA could be relatively recent products of genetic drift. Thus, the primary purpose of this study was to determine the mutation age and conservation of all currently identified human SNPs associated with PA regulation. The outcomes of this study could then be used to initially determine: 1) if significant genomic variants associated with PA were a part of an evolutionarily conserved gene set shared among other species; and 2) if variants emerged at any point in human history that would have required altered engagement in PA due to survival strategies (e.g. hunting/gathering transition to farming)

## Materials and methods

### Overview

The goal of our approach was to combine all published SNPs associated with PA regulation in humans, determine their conservation between humans and closely related species (chimpanzees and Neanderthals), determine conservation among placental mammals, estimate mutation age using two existing prediction databases, and compare current population allele frequencies in Africa, Eastern Asia, and Europe.

### Literature search

To identify the currently known genomic variants associated with PA regulation in humans, the PubMed database was searched on April 20th 2018 with the following combined keywords: (Genome-wide[Title/Abstract] OR SNP[Title/Abstract] OR allele[Title/Abstract]) AND (Physical Activity[Title/Abstract] OR leisure[Title/Abstract] OR sedentary[Title/Abstract] OR exercise participation[Title/Abstract]). Resulting manuscripts were downloaded and placed into a reference management software (Mendeley, INC., New York, NY). Final inclusion was based on a three-step screening process through abstract text and full text when necessary. The inclusion criteria applied were: 1) the study was completed in human populations; 2) PA levels were the primary dependent variable; and 3) the study was not a review of literature. A list of all PA-related SNPs were compiled and searched in the National Center for Biotechnology Information’s SNP Database (https://www.ncbi.nlm.nih.gov/projects/SNP/) to confirm function, location, and allele frequencies.

### Conservation of alleles

To compare conservation of PA-related SNPs with Neanderthal and Chimpanzee genomes, the SNP reference numbers (rs#) were entered into the University of California Santa Cruz Genome Browser (https://genome.ucsc.edu/Neandertal/) which contains data generated by Green et al. [14]. SNPs from five human genomes of diverse ancestries (San, Yoruba, Han, Papuan, and French) and three Neanderthal genomes were labeled as ancestral (A) or derived (D) characterized as such by comparison with the chimpanzee reference genome. Comparisons of genomes were limited to 30% of substitutions and 14% of indels in the human lineage due to incomplete sequencing coverage, disproportionately higher mutation at CpG sites, and a low sample size (n = 3) in the Neanderthal genome. The final database contained 3,202,190 substitutions and 69,029 indels. Additionally, all SNPs were cross-checked with a database created by Bejerano et al. [15] containing 13,736 ultra-conserved elements which were sequences over 100 base pairs that are identical between at least three of five placental mammals. The comparison with the Bejerano database allowed determination of whether the PA-related SNPs emerged in early mammals before human evolution.

### Mutation age prediction

To determine mutation ages, each PA-related SNP was cross-referenced with the National Heart, Lung, and Blood Institute’s Grand Opportunity Exome Sequencing Project (NHLBI GO ESP) Exome Variant Server (http://evs.gs.washington.edu/EVS/) as well as Hawk’s Linkage Disequilibrium Database [16]. The Exome Variant Server contains SNP data from 6,515 unrelated Americans compiled from 20 different cohorts. Mutation origin predictions were produced by Fu et al. [17] using Griffiths and Tavares’s [18] age of mutations in a coalescent tree formula which indicates the origination of gene variants with a common ancestor. This coalescent tree formula was derived from Kimura and Ohta’s [19] formula for calculating the expected age of neutral mutations, variations that are not selected for or against, in a stable population. All simulations were based on the Out-of-Africa Model theorized by Schaffner et al [20] which characterized a bottleneck of non-African populations approximately 51 kya, a second bottleneck for European populations 23 kya, and an accelerated world population growth occurring 5.1 kya. The Linkage Disequilibrium Database contains estimated years since the mutation of 6,509 selected SNPs from the HapMap Project (Northern and Western European ancestry only), which were calculated using rates of linkage disequilibrium decay. This linkage disequilibrium decay indicates the rate at which sections of DNA depart from predictable reshuffling [16]. The use of these techniques produced an estimation of when each PA-related SNP emerged in the human genome as indicated by years before current day.

### Population allele frequency comparisons

To determine the similarity of PA-related SNPs among difference populations, population allele frequencies of Africa, Eastern Asia, and Europe were searched within the 1000 Genomes Project Database using the GRCh37 reference assembly (http://grch37.ensembl.org/Homo_sapiens/Info/Index). Chi-square statistics were used to determine if allele frequencies were significantly different between populations. The alpha level was set at 0.00048 per Bonferroni’s correction for multiple tests.

## Results

### Literature search

The PubMed search yielded 902 results, of which 12 studies met the three-inclusion criterion. Two papers [21,22] were added due to appearance in the literature after the initial search. Of the 14 studies, seven were GWAS and seven were candidate gene studies. Of the seven GWAS, four used SNPs, while all pre-2009 studies utilized micro-satellite technology to estimate Quantitative Trait Loci (QTL). The pre-2009 studies that used micro-satellite technology did not provide specific locations for conservation or age predictions and as a result, were dropped from the analysis. After compiling all results, the eight included studies included reported a total of 104 unique SNPs significantly associated with PA [21–29].

Of the 104 unique SNPs associated with various measurements of PA, six were located in exons, 49 were located in introns, one was in a 3’ UTR region, two were considered upstream of a gene (potential promoter/enhancer), three were considered downstream of a gene (potential transcription unit/terminator), and 43 were intergenic. A comprehensive list of these identified SNPs can be found in supplementary Table 1.

**Table 1:** Human, Neanderthal, and chimpanzee alleles for PA phenotypes.

rsID	Gene	Effect Allele^a^	Neanderthal^b^	Ancestral Allele^c^	Allele Frequencies^d^
rs16933006	Closest RPL7P3	A	AAADAA:0D2A	A>A/C	82/18
rs6025590	CTCFL	A	DAAAA_:0D1A	G>A/G	27/73
rs6454672	CNR1	T	AADAAA:1D0A	T>T/C	85/15
rs8066276	ACE	T	DAD_DA:0D1A	C>C/T	38/62
rs2267668	PPARD	A	DDDDAA:0D1A	A>A/G	85/15
rs1376935	CADM2	G	AAAAAD:0D2A	G>G/A	86/14
rs1638525	AKAP10	G	DADDAD:0D1A	G>C/G	61/39
rs35622985	MMS22L	G	AAAADA:0D1A	G>A/G	27/73
rs1959759	DCAF5	A	ADDDAA:0D2A	A>A/G	18/82
rs10851869	PML	T	D_A_AA:0D1A	C>T/C	57/43
rs2113077	Closest ISL1	A	DAADAD:0D2A	G>A/G	42/58
rs10145335	Closest C14ord177	G	DDDDAD:0D1A	A>G/A	80/20
rs113351744	Closest LINC01029	G	AADAAA:0D1A	G>G/A	98/2
rs12460611	Closest CCNE1	A	AA_DAA:0D3A	A>A/G	83/17
rs12438610	GABRA5	A	ADAAAA:0D1A	G>A/G/T	9/91/.002
rs12595253	GABRG3	A	A_DAAA:0D2A	G>A/G	13/87

^a^ Allele associated with higher amount of physical activity

^b^ First six characters represent the allele present in the following genomes: human reference, San, Yoruba, Han, Papuan, and French. A—ancestral, D—derived, or _ if not known. Following the colon are the amount of derived or ancestral alleles in Neanderthal genomes

^c^ The first character represents the chimpanzee reference allele followed by human alleles

^d^ UCSC allele frequencies of human genome found in previous column

SNPs with no effect allele given in primary study are not listed

### Conservation of alleles

Twenty-nine of the significant PA-related SNPs had chimpanzee/Neanderthal derivations in the University of California Santa Cruz Genome Browser. Of these 29 SNPs, effect alleles were provided in the original articles for 16 SNPs (Table 1). Of those 16 SNPs, the allele associated with a more active phenotype was the most common allele for 10 of 16 alleles in humans, 9 of 16 alleles Neanderthals, and 8 of 16 alleles in chimpanzees. A *post-hoc* analysis using chi-square statistics revealed no significant differences between frequency of “higher PA” associated alleles in humans, Neanderthals, or chimpanzees. However, four PA-related SNPs were predicted to have strong selective sweeps (evidence of strong selection pressure in favor of these alleles) in *Homo sapiens* since their divergence from *Homo neanderthalensis*: rs1051393 exon in *IFNAR2*, rs2267668 intron in *PPARD*, rs1638525 intron of *AKAP10*, and rs10145335 intergenic region near *C14ord177*. There were no matches between PA-related SNPs and the Bejerano et al. database of 13,736 ultra-conserved elements.

### Mutation age prediction

Exon-located SNPs found in *IFNAR2*, *PPARGC1A*, *PML*, *CTBP2*, *IL5RA*, and *APOE* were matched in the NHLBI GO ESP’s Exome Variant Server. *APOE* age was not estimated in the reference database for unknown reasons. For the remaining PA-related SNPs (n = 5), average AA estimated mutation age was 478.4 ± 327.5 kya and average EA mutation age was 542.1 ± 369.4 kya (Table 2). These age ranges can be compared to the average age across all 6,515 SNPs in the Exome Variant Server database of 47.6 ± 1.5 kya in AA and 34.2 ± 0.9 kya in EA. Amongst the non-exonic SNPs, only a SNP located in the intron for *DNAJC1*, rs7910002, was found in the linkage disequilibrium database. The estimated age of selection of this SNP was predicted to be 7.8 kya (Table 2).

**Table 2:** Estimated mutation age of PA-related SNPs.

	African American	European American
All Exons SNPs^a^	47.6 ± 1.5	34.2 ± 0.9
*PPARGC1A*	785.2 ± 414.7	666.0 ± 402.3
*IFNAR2*	747.1 ± 411.8	681.0 ± 403.7
*IL-15Ra*	585.2 ± 391.8	427.4 ± 341.5
*PML*	221.7 ± 238.7	549.1 ± 385.5
*CTBP2*	210.5 ± 231	229.1 ± 263 .9
*DNAJC1*		7.8
**Average Exon**	**478.4 ± 327.5**	**542.1 ± 369.4**

Predictions are listed as predicted mutation age in thousands of years ago ± range

^a^All exon located SNPs predicted by Fu et al. (12)

### Population allele frequency comparisons

**Fig 1:**
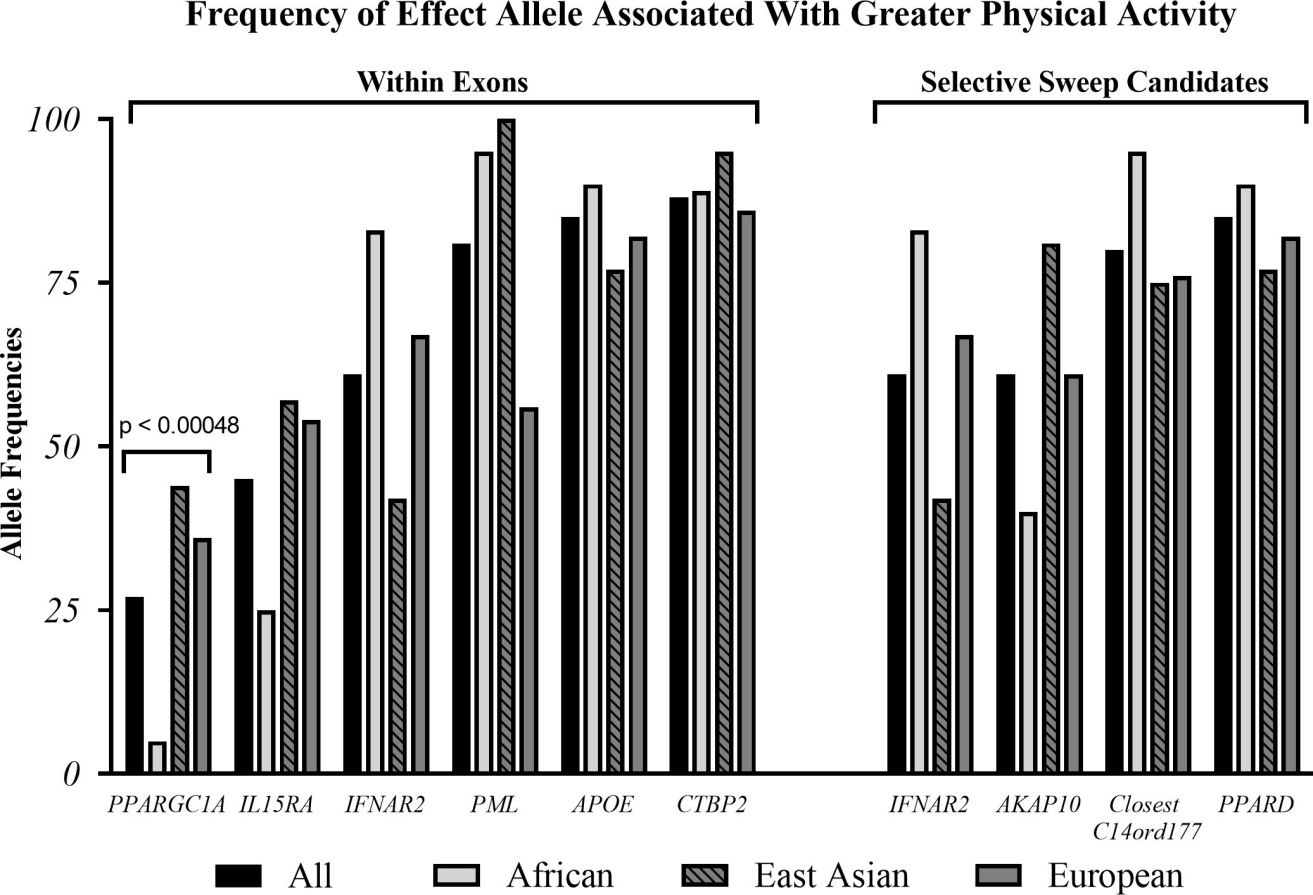
Effect allele frequencies of african, eastern asian, and european populations. Chi-square statistics indicated a significant difference between population allele frequencies in *PPARGC1A*. All other SNPs located in exons or were strong candidates for selective sweeps had non-significant differences in population allele frequencies.

Of the 104 chi-square tests ran, 38% of the PA-related SNPs had at least one population, African, Eastern Asian, or European, with significantly different allele frequencies. A list of all the tests can be found in S1 Supporting Information. Of the six SNPs within exons and four SNPs who are strong candidates of selective sweeps, there was only a significant difference between population frequencies in the *PPARGC1A* gene (Fig 1).

## Discussion

**Fig 2:**
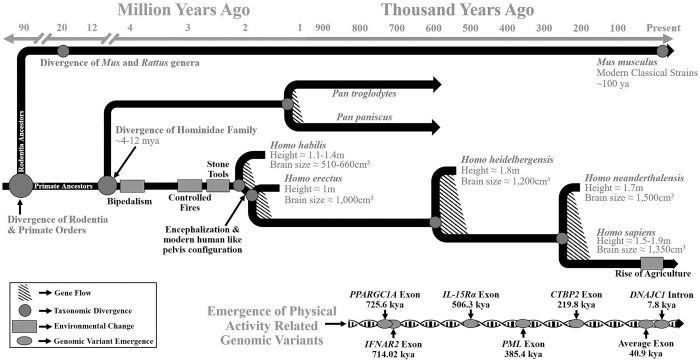
The emergence of PA-Related SNPs and potential selection pressures. PA-related SNPs in protein-coding genomic areas are as old or older than the hypothesized emergence of anatomically modern humans (~200–350 kya). The only intron (*DNAJC1*) was predicted to emerge around the time of the rise in agriculture.

The present study is the first to attempt dating the currently known human-related genomic sequence variations associated with PA in an attempt to further understand the origin of PA regulation and its conservation among species. In general, our results estimate PA-related SNPs in protein-coding genomic areas are as old or older than the hypothesized emergence of anatomically modern humans (~200–350 kya) [30–32]. This estimated SNP age range is considerably older than the majority of exon-located mutations given that Fu et al. predicted 73.2% of modern day exonal SNPs arose less than 5 kya [17]. A mutation age range from 210 to 785 kya suggests the genetic control of PA in humans, through these particular exon-located SNPs, may have emerged during periods of *Homo* energy expenditure alterations such as the dramatic increase in *Homo*’s brain size, body size, and/or gathering range [30,31]. A proposed timeline equating the emergence of PA associated mutations with possible selection pressures is in Fig 2. Given the evolutionary divergence of humans and primates has been approximated to be 4–12 mya [33,34], our species may not share the exact intragenic-based modulators of PA present in primates. Further, while many PA genetic studies are completed in mice models, the evolutionary split between humans and mice is estimated to have occurred approximately 96 mya similarly suggesting intragenic-based modulators controlling activity in humans may not be similar to those controlling activity in mice [33–35]. However, we urge caution in interpreting our limited dataset to mean there are no similarities among human PA genetic regulation and PA regulation in other species given that the direct causal link between the available SNPs and PA is yet to be determined.

Further supporting our estimated mutation age of the PA-related SNPs, there were no matches between PA-related SNPs and the database of 13,736 ultra-conserved elements [15]. Thus, there is little to no known conservation of any PA-related SNPs in humans among other earlier mammalian placental species, suggesting that the PA-related SNPs emerged after the split of *Homo erectus* from other mammals (Fig 2). Comparison of the 16 SNPs where Neanderthal and chimpanzee genome data were available revealed the effect allele for “more physical activity” was possessed by the majority of humans 63% of the time and 56% of the time for sequenced Neanderthals. The chimpanzee reference genome had the effect allele as the primary allele 50% of the time. Statistical analysis determined these differences could be accredited to chance alone, indicating no clear genetic basis for any species as “more active”. Thus, this genetic data does not explain why chimpanzee daily PA is 3–6 times lower than modern day hunter-gatherers [36–38].

The four selective sweeps in the *Homo sapiens* lineage reveals at least a partial divergence from *Homo neanderthalensis* with increased fitness at these specific locations. Mutations that altered the amount of PA could have improved fitness by increasing the motivation for movement. For example, there is evidence for increased ranging size in early *Homo* relatives around 2 mya that laziness would have directly decreased fitness [39]. However, the split between *Homo sapiens and Homo neanderthalensis* is estimated to have occurred much later (~200–350 kya). It is possible the altered regulation of PA could have been a secondary phenotype as gene functions of the four selective sweeps have no clear connection with PA regulation. The protein coded by *IFNAR2* forms a part of membrane receptor for interferons eventually leading to the phosphorylation of many proteins typically associated with infection prevention. Knockouts of the *PPARD* gene suggest a role in myelination of the corpus callosum as well as lipid metabolism. *AKAP10* is known to confine regulatory subunits of protein kinase A to discrete regions of mitochondria. Polymorphisms within *AKAP10* have been associated with increased risk of arrythmias and sudden cardiac death. rs1014533 is found intergenically located closest to *C14orf177* [40]. It is unknown if this SNP is connected to this particular open reading frame but would likely suggest a role in gene expression. The suggestions that genetic factors regulate PA as a secondary phenotype is supported by the fact other PA-related SNPs that were not included in this aging analysis due to location outside of coding areas or lack of age-estimation data have been related to various other phenotypes such as obesity, sensation seeking, depression, blood flow, mitochondrial function, inflammation, Alzheimer’s, blood lipid levels, metabolic syndrome, and height. A list of all known phenotypes associated with each SNP and gene can be found in S1 Supporting Information.

Of the 104 PA-related SNPs, only 38% of the SNPs had at least one population (African, Eastern Asian, or European) with a significantly unique allele frequency. The largest disparity was found between African and European populations in rs1993246, an intron of *KCCATT33*, where the C allele is 60% more common in Europeans. Due to the large disparity, this allele is likely to have emerged after the splitting of these two populations. Other than rs1993246 and rs8192678, an exon in the *PPARGC1A* gene (Fig 1), the generally low disparity in the other allele frequencies between the populations may indicate at least some conservation within human populations since initial mutation development and growth.

Predictions of the emergence and conservation of PA-related SNPs are complicated by assuming these mutations improved fitness as mutations aged 400–500 kya would likely be fixed in current day, but the data suggest these mutations are not currently fixed. It is possible genetic mutations altering rates of PA were dependent on the environment and were not always advantageous throughout time or between groups. For example, methods of obtaining food are different depending on region, climate, and innovations. While selection may have occurred during periods of time 400–500 kya, genetic drift may have played the strongest effect on allele frequencies in recent years.

A limitation of this paper is only six of 104 unique human PA-related SNPs found to date are located in protein-coding exons. The large number of PA-related non-exon located SNPs raises the question whether these SNPs have any PA regulation function or if they are significant due to random chance alone. Previous papers in rodent models have observed a similar percentage of PA-related SNPs in intergenic areas [41] as well as sets of micro-RNAs associated with high- and low-activity mice [42]. The available databases only enable the age estimation of one of the human PA-related intron SNPs with the age estimated for this intron SNP (7,796 kya) being much younger than those we observed from the exon SNPs. The age of this intron SNP (from the *DNAJC1* gene), is close to the beginning of the onset of farming in humans [12]. While we will make no speculation of how the *DNAJC1* mutation may have improved fitness and rose in frequency, it is possible that with further dating of the other activity-associated SNPs a more comprehensive timeline of the evolution of the genetic mechanisms regulating PA can be developed. Further limiting our predictions, SNPs with larger minor allele frequencies are more likely to be discovered via GWAS. Since the method by Fu et al. uses allele frequency as the heaviest weighted factor to determine mutation age, GWAS discovered SNPs are expected to be predicted as older than the average SNP.

In summary, our results show that where the age-estimation data is available, exon-located SNPs that are associated with the regulation of PA in humans arose between 210 and 785 kya while intergenic SNPs may be much younger. The estimations at this point represent just a small fraction of the known PA-related SNPs, but provide an initial framework for better understanding the origins of PA genetic regulation. Further resolution of this evolutionary timeline will require additional studies and an understanding of the role genomic factors located outside of the protein-coding sequences play in the regulation of PA.

## Supporting information

S1 Supporting Information (XLSX)
